# 1,1′-Methyl­enebis(4-*tert*-butyl­pyridinium) dichloride hemihydrate

**DOI:** 10.1107/S2414314621007689

**Published:** 2021-08-03

**Authors:** J.P.J. Bruekers, J.A.A.W. Elemans, R.J.M. Nolte, P. Tinnemans

**Affiliations:** a Radboud University, Institute for Molecules and Materials, Heyendaalseweg 135, 6525 AJ, Nijmegen, The Netherlands; Goethe-Universität Frankfurt, Germany

**Keywords:** crystal structure, O—H⋯Cl hydrogen bonds, Cl⋯π contacts

## Abstract

The structure of the title hydrated salt, C_19_H_28_N_2_^2+^·2Cl^−^·0.5H_2_O, at 150 K has monoclinic (*C*2/*c*) symmetry. The water mol­ecule is located on a twofold rotation axis.

## Abstract 2

The title compound, C_19_H_28_N_2_^2+^·2Cl^−^·0.5H_2_O, was prepared by the reaction of 4-*tert*-butyl­pyridine with di­chloro­methane. One of the chloride anions is clamped between the aromatic rings *via* anion–π inter­actions.

## Graphical abstract

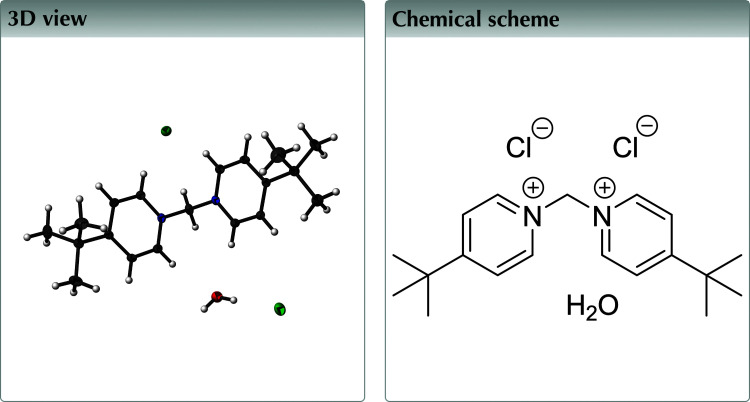

## Structure description

**Figure 1:**
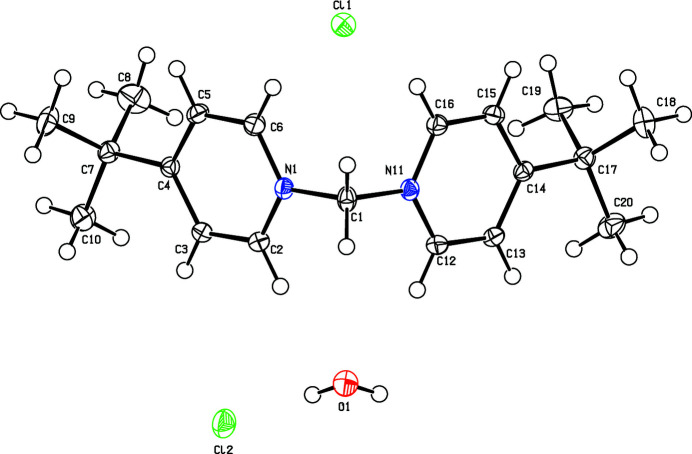
Mol­ecular structure of the title compound with atom labels. Displacement ellipsoids are at the 50% probability level.

**Figure 2:**
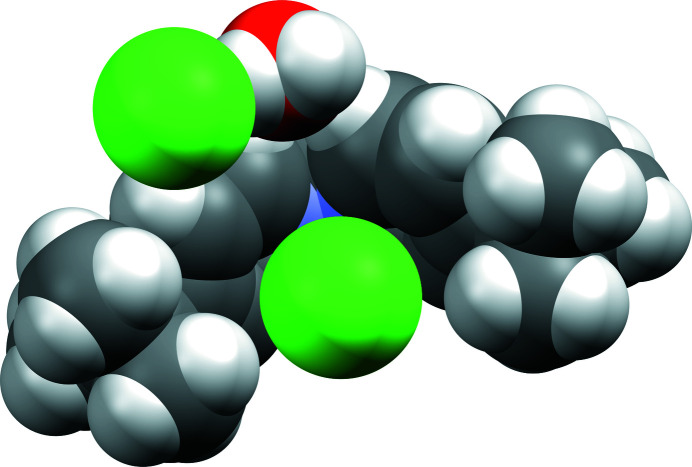
Space-filling representation of the title compound showing one chloride hydrogen bonded to the water mol­ecule and a chloride ion clamped between the aromatic rings.

The title compound (Fig. 1 ▸) was prepared before by mixing 4-*tert*-butyl­pyridine with di­chloro­methane in DMSO (Rudine *et al.*, 2010 ▸). The crystal structure of a related compound, 1,1′-methyl­enebis(4-*tert*-butyl­pyridinium)chlorido­cobaltate(II)–di­chloro­methane (1:1), has been determined (Ayom *et al.*, 2019 ▸). The di-cation of the title compound has a V-shaped structure caused by the bridging methyl­ene group with an N—C—N angle of 109.30 (10)°. One of the chloride anions forms a hydrogen bond with the water mol­ecule (Table 1 ▸). The other chloride anion is clamped between the aromatic rings (Fig. 2 ▸) by electrostatic and anion–π inter­actions with distances to the centroid of the mean planes through the pyridinium rings of 3.3907 (6) and 3.4135 (6) Å, which are similar to the distances of anion–π inter­actions in the literature (Kan *et al.*, 2018 ▸; Demeshko *et al.*, 2004 ▸).

## Synthesis and crystallization

The title compound was obtained during an attempt to grow single crystals of 4-*tert*-butyl­pyridine coordinated to a cadmium derivative of a porphyrin di­phenyl­glycoluril cage reported by Gilissen *et al.* (2019 ▸) by slow evaporation from a 4-*tert*-butyl­pyridine/di­chloro­methane/heptane (1:2:2, *v*/*v*/*v*) mixture. The mixture was left at 298 K. Colorless needle-shaped crystals were obtained after one week.

## Refinement

Crystal data, data collection and structure refinement details are summarized in Table 2 ▸.

## Supplementary Material

Crystal structure: contains datablock(s) I. DOI: 10.1107/S2414314621007689/bt4117sup1.cif

Structure factors: contains datablock(s) I. DOI: 10.1107/S2414314621007689/bt4117Isup2.hkl

Supporting information file. DOI: 10.1107/S2414314621007689/bt4117Isup3.cml

CCDC reference: 2099687

Additional supporting information:  crystallographic information; 3D view; checkCIF report

## Figures and Tables

**Table 1:** Hydrogen-bond geometry (Å, °)

*D*—H⋯*A*	*D*—H	H⋯*A*	*D*⋯*A*	*D*—H⋯*A*
O1—H1⋯Cl2^i^	0.829 (19)	2.223 (19)	3.0513 (10)	179 (2)

Symmetry code: (i) 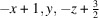 .

**Table 2:** Experimental details

Crystal data	
Chemical formula	C_19_H_28_N_2_^2+^·2Cl^−^·0.5H_2_O
*M*_r_	364.34
Crystal system, space group	Monoclinic, *C*2/*c*
Temperature (K)	150
*a*, *b*, *c* (Å)	27.7659 (8), 5.9001 (2), 23.9758 (7)
β (°)	97.4152 (14)
*V* (Å^3^)	3894.9 (2)
*Z*	8
Radiation type	Mo *K*α
μ (mm^−1^)	0.34
Crystal size (mm)	0.55 × 0.14 × 0.03

Data collection	
Diffractometer	Bruker D8 Quest APEX3
Absorption correction	Multi-scan (*SADABS*; Krause *et al.*, 2015 ▸)
*T*_min_, *T*_max_	0.601, 0.747
No. of measured, independent and observed [*I* > 2σ(*I*)] reflections	36472, 7428, 5988
*R*_int_	0.041
(sin θ/λ)_max_ (Å^−1^)	0.770

Refinement	
*R*[*F* ^2^ > 2σ(*F* ^2^)], *wR*(*F* ^2^), *S*	0.049, 0.106, 1.09
No. of reflections	7428
No. of parameters	222
H-atom treatment	H atoms treated by a mixture of independent and constrained refinement
Δρ_max_, Δρ_min_ (e Å^−3^)	0.47, −0.34

Computer programs: *APEX3* (Bruker, 2017 ▸), *SAINT* (Bruker, 2003 ▸), *SHELXT2014/5* (Sheldrick, 2015*a* ▸), *SHELXL2018/3* (Sheldrick, 2015*b* ▸), *PLATON* (Spek, 2020 ▸) and *shelXle* (Hübschle *et al.*, 2011 ▸).

## full crystallographic data

### Crystal data

**Table:**

C_19_H_28_N_2_^2+^·2Cl^−^·0.5H_2_O	*F*(000) = 1560
*M**_r_* = 364.34	*D*_x_ = 1.243 Mg m^−^^3^
Monoclinic, *C*2/*c*	Mo *K*α radiation, λ = 0.71073 Å
*a* = 27.7659 (8) Å	Cell parameters from 9986 reflections
*b* = 5.9001 (2) Å	θ = 2.4–32.9°
*c* = 23.9758 (7) Å	µ = 0.34 mm^−^^1^
β = 97.4152 (14)°	*T* = 150 K
*V* = 3894.9 (2) Å^3^	Needle, colourless
*Z* = 8	0.55 × 0.14 × 0.03 mm

### Data collection

**Table:**

Bruker D8 Quest APEX3 diffractometer	7428 independent reflections
Radiation source: sealed tube	5988 reflections with *I* > 2σ(*I*)
Graphite monochromator	*R*_int_ = 0.041
Detector resolution: 7.41 pixels mm^-1^	θ_max_ = 33.2°, θ_min_ = 2.4°
φ and ω scans	*h* = −40→42
Absorption correction: multi-scan (SADABS; Krause *et al.*, 2015)	*k* = −9→7
*T*_min_ = 0.601, *T*_max_ = 0.747	*l* = −36→36
36472 measured reflections	

### Refinement

**Table:**

Refinement on *F*^2^	Primary atom site location: structure-invariant direct methods
Least-squares matrix: full	Secondary atom site location: difference Fourier map
*R*[*F*^2^ > 2σ(*F*^2^)] = 0.049	Hydrogen site location: mixed
*wR*(*F*^2^) = 0.106	H atoms treated by a mixture of independent and constrained refinement
*S* = 1.09	*w* = 1/[σ^2^(*F*_o_^2^) + (0.0345*P*)^2^ + 4.7462*P*] where *P* = (*F*_o_^2^ + 2*F*_c_^2^)/3
7428 reflections	(Δ/σ)_max_ = 0.002
222 parameters	Δρ_max_ = 0.47 e Å^−^^3^
0 restraints	Δρ_min_ = −0.34 e Å^−^^3^

### Special details

**Table:**

Geometry. All esds (except the esd in the dihedral angle between two l.s. planes) are estimated using the full covariance matrix. The cell esds are taken into account individually in the estimation of esds in distances, angles and torsion angles; correlations between esds in cell parameters are only used when they are defined by crystal symmetry. An approximate (isotropic) treatment of cell esds is used for estimating esds involving l.s. planes.
Refinement. Non-hydrogen atoms were refined freely with anisotropic displacement parameters. Hydrogen atoms were placed on calculated positions or located in difference Fourier maps. Hydrogen atoms bonded to C were refined with a riding model with U(H)= 1.2*U*_eq_(C_aromatic_) or *U*(H)= 1.5*U*_eq_(C_methyl_). The coordinates of the H atom bonded to O were refined with *U*(H)= 1.5*U*_eq_(O).

### Fractional atomic coordinates and isotropic or equivalent isotropic displacement parameters (Å^2^)

**Table:**

	*x*	*y*	*z*	*U*_iso_*/*U*_eq_	
Cl1	0.66762 (2)	1.24711 (5)	0.69007 (2)	0.02011 (7)	
Cl2	0.46510 (2)	−0.04518 (7)	0.65504 (2)	0.02807 (9)	
O1	0.500000	0.2834 (2)	0.750000	0.0226 (3)	
H1	0.5097 (7)	0.194 (3)	0.7757 (8)	0.034*	
N1	0.58269 (4)	0.70318 (18)	0.66729 (4)	0.01528 (19)	
C1	0.58379 (5)	0.8164 (2)	0.72238 (5)	0.0183 (2)	
H1A	0.594236	0.975901	0.719446	0.022*	
H1B	0.550893	0.815733	0.734115	0.022*	
C2	0.55369 (5)	0.5221 (2)	0.65514 (5)	0.0184 (2)	
H2	0.533083	0.472634	0.681422	0.022*	
C3	0.55359 (5)	0.4077 (2)	0.60496 (5)	0.0178 (2)	
H3	0.532917	0.280414	0.596878	0.021*	
C4	0.58376 (4)	0.4784 (2)	0.56596 (5)	0.0147 (2)	
C5	0.61278 (5)	0.6685 (2)	0.58025 (5)	0.0185 (2)	
H5	0.633346	0.723036	0.554488	0.022*	
C6	0.61224 (5)	0.7782 (2)	0.63070 (5)	0.0183 (2)	
H6	0.632542	0.906011	0.639799	0.022*	
C7	0.58699 (5)	0.3558 (2)	0.51056 (5)	0.0178 (2)	
C8	0.63866 (6)	0.2601 (3)	0.51317 (8)	0.0325 (3)	
H8A	0.641469	0.173104	0.478916	0.049*	
H8B	0.645413	0.160829	0.546031	0.049*	
H8C	0.662081	0.385129	0.516240	0.049*	
C9	0.57717 (6)	0.5233 (3)	0.46124 (6)	0.0272 (3)	
H9A	0.579490	0.443658	0.425820	0.041*	
H9B	0.601237	0.645579	0.465983	0.041*	
H9C	0.544529	0.587349	0.460507	0.041*	
C10	0.55031 (6)	0.1622 (3)	0.50068 (6)	0.0261 (3)	
H10A	0.517323	0.222912	0.499241	0.039*	
H10B	0.556244	0.052461	0.531479	0.039*	
H10C	0.553860	0.087118	0.464978	0.039*	
N11	0.61792 (4)	0.69581 (19)	0.76441 (4)	0.01542 (19)	
C12	0.60175 (5)	0.5233 (2)	0.79374 (5)	0.0185 (2)	
H12	0.568214	0.485537	0.788490	0.022*	
C13	0.63333 (5)	0.4013 (2)	0.83121 (5)	0.0178 (2)	
H13	0.621456	0.280122	0.851674	0.021*	
C14	0.68271 (4)	0.4544 (2)	0.83935 (5)	0.0149 (2)	
C15	0.69812 (4)	0.6362 (2)	0.80819 (5)	0.0169 (2)	
H15	0.731437	0.678077	0.812838	0.020*	
C16	0.66551 (5)	0.7540 (2)	0.77110 (5)	0.0173 (2)	
H16	0.676331	0.876209	0.750120	0.021*	
C17	0.71984 (5)	0.3212 (2)	0.87856 (5)	0.0179 (2)	
C18	0.74794 (6)	0.4840 (3)	0.92118 (6)	0.0267 (3)	
H18A	0.764762	0.597771	0.901042	0.040*	
H18B	0.771764	0.398124	0.946550	0.040*	
H18C	0.725171	0.559656	0.943085	0.040*	
C19	0.75542 (5)	0.2126 (2)	0.84213 (6)	0.0245 (3)	
H19A	0.737128	0.126977	0.811366	0.037*	
H19B	0.777406	0.110051	0.865328	0.037*	
H19C	0.774353	0.331603	0.826525	0.037*	
C20	0.69621 (5)	0.1354 (3)	0.91046 (6)	0.0262 (3)	
H20A	0.678134	0.031685	0.883493	0.039*	
H20B	0.673927	0.204551	0.934073	0.039*	
H20C	0.721457	0.051023	0.934211	0.039*	

### Atomic displacement parameters (Å^2^)

**Table:**

	*U*^11^	*U*^22^	*U*^33^	*U*^12^	*U*^13^	*U*^23^
Cl1	0.02257 (14)	0.01460 (13)	0.02306 (14)	−0.00075 (11)	0.00262 (10)	−0.00094 (11)
Cl2	0.03340 (18)	0.03155 (18)	0.01914 (14)	−0.00159 (15)	0.00295 (12)	−0.00336 (13)
O1	0.0265 (7)	0.0178 (7)	0.0234 (7)	0.000	0.0025 (5)	0.000
N1	0.0157 (4)	0.0171 (5)	0.0130 (4)	0.0015 (4)	0.0012 (3)	−0.0009 (3)
C1	0.0191 (5)	0.0215 (6)	0.0139 (5)	0.0059 (5)	0.0006 (4)	−0.0027 (4)
C2	0.0163 (5)	0.0236 (6)	0.0159 (5)	−0.0028 (5)	0.0035 (4)	0.0004 (4)
C3	0.0168 (5)	0.0202 (6)	0.0161 (5)	−0.0044 (5)	0.0012 (4)	0.0002 (4)
C4	0.0148 (5)	0.0153 (5)	0.0139 (5)	0.0010 (4)	0.0011 (4)	0.0011 (4)
C5	0.0212 (6)	0.0192 (6)	0.0160 (5)	−0.0053 (5)	0.0055 (4)	−0.0002 (4)
C6	0.0217 (6)	0.0161 (6)	0.0170 (5)	−0.0033 (5)	0.0019 (4)	−0.0003 (4)
C7	0.0203 (6)	0.0171 (6)	0.0165 (5)	−0.0002 (5)	0.0044 (4)	−0.0023 (4)
C8	0.0264 (7)	0.0316 (8)	0.0410 (8)	0.0069 (6)	0.0094 (6)	−0.0095 (7)
C9	0.0396 (8)	0.0274 (7)	0.0148 (5)	−0.0023 (6)	0.0043 (5)	0.0002 (5)
C10	0.0337 (7)	0.0226 (7)	0.0222 (6)	−0.0074 (6)	0.0045 (5)	−0.0071 (5)
N11	0.0156 (4)	0.0179 (5)	0.0128 (4)	0.0015 (4)	0.0017 (3)	−0.0020 (4)
C12	0.0150 (5)	0.0236 (6)	0.0173 (5)	−0.0023 (5)	0.0039 (4)	−0.0005 (5)
C13	0.0184 (5)	0.0196 (6)	0.0159 (5)	−0.0031 (5)	0.0047 (4)	0.0016 (4)
C14	0.0168 (5)	0.0148 (5)	0.0136 (5)	0.0000 (4)	0.0039 (4)	−0.0013 (4)
C15	0.0151 (5)	0.0174 (6)	0.0183 (5)	−0.0019 (4)	0.0026 (4)	−0.0004 (4)
C16	0.0186 (5)	0.0173 (5)	0.0162 (5)	−0.0025 (5)	0.0026 (4)	−0.0002 (4)
C17	0.0188 (5)	0.0171 (5)	0.0178 (5)	0.0019 (4)	0.0024 (4)	0.0018 (4)
C18	0.0298 (7)	0.0269 (7)	0.0212 (6)	0.0023 (6)	−0.0051 (5)	−0.0021 (5)
C19	0.0223 (6)	0.0213 (7)	0.0313 (7)	0.0042 (5)	0.0087 (5)	−0.0002 (5)
C20	0.0272 (7)	0.0249 (7)	0.0273 (7)	0.0038 (6)	0.0065 (5)	0.0108 (6)

### Geometric parameters (Å, º)

**Table:**

O1—H1	0.829 (19)	C10—H10A	0.9800
O1—H1^i^	0.829 (19)	C10—H10B	0.9800
N1—C2	1.3468 (17)	C10—H10C	0.9800
N1—C6	1.3509 (16)	N11—C12	1.3465 (17)
N1—C1	1.4768 (16)	N11—C16	1.3547 (16)
C1—N11	1.4740 (16)	C12—C13	1.3749 (18)
C1—H1A	0.9900	C12—H12	0.9500
C1—H1B	0.9900	C13—C14	1.3955 (17)
C2—C3	1.3792 (18)	C13—H13	0.9500
C2—H2	0.9500	C14—C15	1.4048 (17)
C3—C4	1.3972 (17)	C14—C17	1.5213 (18)
C3—H3	0.9500	C15—C16	1.3732 (18)
C4—C5	1.3976 (18)	C15—H15	0.9500
C4—C7	1.5249 (17)	C16—H16	0.9500
C5—C6	1.3735 (18)	C17—C20	1.5320 (19)
C5—H5	0.9500	C17—C18	1.539 (2)
C6—H6	0.9500	C17—C19	1.5402 (19)
C7—C10	1.528 (2)	C18—H18A	0.9800
C7—C8	1.536 (2)	C18—H18B	0.9800
C7—C9	1.5379 (19)	C18—H18C	0.9800
C8—H8A	0.9800	C19—H19A	0.9800
C8—H8B	0.9800	C19—H19B	0.9800
C8—H8C	0.9800	C19—H19C	0.9800
C9—H9A	0.9800	C20—H20A	0.9800
C9—H9B	0.9800	C20—H20B	0.9800
C9—H9C	0.9800	C20—H20C	0.9800
			
H1—O1—H1^i^	101 (3)	C7—C10—H10C	109.5
C2—N1—C6	121.04 (11)	H10A—C10—H10C	109.5
C2—N1—C1	119.67 (11)	H10B—C10—H10C	109.5
C6—N1—C1	119.24 (11)	C12—N11—C16	121.02 (11)
N11—C1—N1	109.30 (10)	C12—N11—C1	119.58 (11)
N11—C1—H1A	109.8	C16—N11—C1	119.33 (11)
N1—C1—H1A	109.8	N11—C12—C13	120.63 (11)
N11—C1—H1B	109.8	N11—C12—H12	119.7
N1—C1—H1B	109.8	C13—C12—H12	119.7
H1A—C1—H1B	108.3	C12—C13—C14	120.39 (12)
N1—C2—C3	120.71 (11)	C12—C13—H13	119.8
N1—C2—H2	119.6	C14—C13—H13	119.8
C3—C2—H2	119.6	C13—C14—C15	117.28 (11)
C2—C3—C4	120.27 (12)	C13—C14—C17	123.06 (11)
C2—C3—H3	119.9	C15—C14—C17	119.63 (11)
C4—C3—H3	119.9	C16—C15—C14	120.66 (11)
C3—C4—C5	116.85 (11)	C16—C15—H15	119.7
C3—C4—C7	123.32 (11)	C14—C15—H15	119.7
C5—C4—C7	119.82 (11)	N11—C16—C15	120.01 (12)
C6—C5—C4	121.52 (11)	N11—C16—H16	120.0
C6—C5—H5	119.2	C15—C16—H16	120.0
C4—C5—H5	119.2	C14—C17—C20	112.25 (11)
N1—C6—C5	119.61 (12)	C14—C17—C18	109.44 (11)
N1—C6—H6	120.2	C20—C17—C18	109.15 (11)
C5—C6—H6	120.2	C14—C17—C19	107.32 (10)
C4—C7—C10	112.12 (10)	C20—C17—C19	109.42 (12)
C4—C7—C8	107.37 (11)	C18—C17—C19	109.20 (12)
C10—C7—C8	109.36 (12)	C17—C18—H18A	109.5
C4—C7—C9	109.91 (11)	C17—C18—H18B	109.5
C10—C7—C9	108.22 (12)	H18A—C18—H18B	109.5
C8—C7—C9	109.86 (12)	C17—C18—H18C	109.5
C7—C8—H8A	109.5	H18A—C18—H18C	109.5
C7—C8—H8B	109.5	H18B—C18—H18C	109.5
H8A—C8—H8B	109.5	C17—C19—H19A	109.5
C7—C8—H8C	109.5	C17—C19—H19B	109.5
H8A—C8—H8C	109.5	H19A—C19—H19B	109.5
H8B—C8—H8C	109.5	C17—C19—H19C	109.5
C7—C9—H9A	109.5	H19A—C19—H19C	109.5
C7—C9—H9B	109.5	H19B—C19—H19C	109.5
H9A—C9—H9B	109.5	C17—C20—H20A	109.5
C7—C9—H9C	109.5	C17—C20—H20B	109.5
H9A—C9—H9C	109.5	H20A—C20—H20B	109.5
H9B—C9—H9C	109.5	C17—C20—H20C	109.5
C7—C10—H10A	109.5	H20A—C20—H20C	109.5
C7—C10—H10B	109.5	H20B—C20—H20C	109.5
H10A—C10—H10B	109.5		
			
C2—N1—C1—N11	84.05 (14)	N1—C1—N11—C12	−89.45 (13)
C6—N1—C1—N11	−93.47 (14)	N1—C1—N11—C16	87.62 (14)
C6—N1—C2—C3	0.26 (19)	C16—N11—C12—C13	−0.16 (19)
C1—N1—C2—C3	−177.21 (12)	C1—N11—C12—C13	176.86 (11)
N1—C2—C3—C4	0.1 (2)	N11—C12—C13—C14	−0.08 (19)
C2—C3—C4—C5	−0.76 (19)	C12—C13—C14—C15	0.39 (18)
C2—C3—C4—C7	177.78 (12)	C12—C13—C14—C17	−177.78 (12)
C3—C4—C5—C6	1.09 (19)	C13—C14—C15—C16	−0.48 (18)
C7—C4—C5—C6	−177.51 (12)	C17—C14—C15—C16	177.75 (12)
C2—N1—C6—C5	0.06 (19)	C12—N11—C16—C15	0.07 (18)
C1—N1—C6—C5	177.54 (12)	C1—N11—C16—C15	−176.96 (11)
C4—C5—C6—N1	−0.8 (2)	C14—C15—C16—N11	0.26 (19)
C3—C4—C7—C10	5.16 (18)	C13—C14—C17—C20	−3.89 (17)
C5—C4—C7—C10	−176.34 (12)	C15—C14—C17—C20	177.98 (12)
C3—C4—C7—C8	−114.97 (14)	C13—C14—C17—C18	−125.25 (13)
C5—C4—C7—C8	63.53 (16)	C15—C14—C17—C18	56.62 (15)
C3—C4—C7—C9	125.57 (13)	C13—C14—C17—C19	116.37 (13)
C5—C4—C7—C9	−55.93 (16)	C15—C14—C17—C19	−61.76 (15)

Symmetry code: (i) −*x*+1, *y*, −*z*+3/2.

### Hydrogen-bond geometry (Å, º)

**Table:**

*D*—H···*A*	*D*—H	H···*A*	*D*···*A*	*D*—H···*A*
O1—H1···Cl2^i^	0.829 (19)	2.223 (19)	3.0513 (10)	179 (2)

Symmetry code: (i) −*x*+1, *y*, −*z*+3/2.
